# Initialization by a Novel Clustering for Wavelet Neural Network as Time Series Predictor

**DOI:** 10.1155/2015/572592

**Published:** 2015-04-22

**Authors:** Rong Cheng, Hongping Hu, Xiuhui Tan, Yanping Bai

**Affiliations:** ^1^School of Science, North University of China, Shanxi, Taiyuan 030051, China; ^2^School of Information and Communication Engineering, North University of China, Shanxi, Taiyuan 030051, China

## Abstract

The architecture and parameter initialization of wavelet neural network are discussed and a novel initialization method is proposed. The new approach can be regarded as a dynamic clustering procedure which will derive the neuron number as well as the initial value of translation and dilation parameters according to the input patterns and the activating wavelets functions. Three simulation examples are given to examine the performance of our method as well as Zhang's heuristic initialization approach. The results show that the new approach not only can decide the WNN structure automatically, but also provides superior initial parameter values that make the optimization process more stable and quickly.

## 1. Introduction

An artificial neural network (ANN) is a highly parallel distributed network of connected processing units called neurons. Due to their fascinating characteristics of robustness, fault tolerance, adaptive learning ability, and massive parallel processing capabilities, ANNs possess the capability of learning from examples with both linear and nonlinear relationships between the input and output signals, which makes them a popular tool for time series prediction [1, 2], feature extraction [3, 4], pattern recognition [5, 6], and classification [7, 8]. However, ANNs have limited ability to characterize local features, such as discontinuities in curvature, jumps in value, or other edges.

Instead of using common sigmoid activation functions, the wavelet neural network (WNN) employing nonlinear wavelet basis functions [9, 10], which are localized in both the time space and frequency space, has been developed as an alternative approach to nonlinear fitting problem. It has been proven that families of wavelet frames are universal approximators [11], which give a theoretical basis to their use in the framework of function approximation and process modeling.

There are two different WNN architectures: one type has fixed wavelet bases possessing fixed dilation and translation parameters (WNN-Type1). In this one only the output layer weights are adjustable. Another type has the variable wavelet base whose dilation and translation parameters and output layer weights are adjustable (WNN-Type2). Several WNN models have been proposed in the literatures. In [12], a four-layer self-constructing wavelet network (SCWN) controller for nonlinear systems control is described and the orthogonal wavelet functions are adopted as its node functions. In [13], a local linear wavelet neural network (LLWNN) is presented whose connection weights between the hidden layer and output layer of conventional WNN are replaced by a local linear model. In [14], a model of multiwavelet-based neural networks is proposed. The structure of this network is similar to that of the wavelet network, except that the orthonormal scaling functions are replaced by orthonormal multiscaling functions.

A time series is a sequence of observations taken sequentially in time [15]. Time series prediction is an important research and application area. Much effort has been devoted over the past several decades to the development and improvement of time series prediction models. Besides the well-known linear models such as moving average, exponential smoothing, and the autoregressive integrated moving average, nonlinear models including artificial neural network, wavelet neural network, and fuzzy system models also become the well-established time series models. In this paper, the wavelet neural network (WNN) is used as the time series predictor, and the detailed research works are described subsequently.

We adopt WNN-Type2 with adjustable translation and dilation parameters and multiplication form of multidimensional wavelets as the nonlinear model for time series prediction in this paper. Key problems in designing of this type of WNN consist of determining WNN architecture, initializing the translation and dilation vectors, and choosing learning algorithm that can be effectively used for training the WNN. This study mainly focuses on the first two points. In the practical applications, the number of hidden neurons which determines the structure of the network is often set by experience or the time-consuming trial-and-error tests, and the initial values of parameters are often set randomly. Due to the rapidly vanishing property of wavelet functions, the random initialization scheme to the dilation and translation parameters may cause the wavelets' effective response regions out of interest which makes the learning performance very instable. So it is inadvisable to adopt random initialization scheme for dilations and translations in WNN. In [9], Zhang proposes a heuristic initialization procedure which considers the interesting domain of input patterns. But, in its implementation, the wavelet functions used in WNN are not considered, and the resolution reduced gradually according to an established rule which does not take full consideration of sample distribution.

In the present paper, inspired by the localization character of wavelet functions and considering the multiplication form of multidimensional wavelets in the hidden neuron for multivariable inputs, we present a novel initialization approach by the help of a new clustering method for WNN. This approach can determine the unit number of hidden layer and initialize the translation and dilation vectors simultaneously. After performing the training process by gradient descent method, we can see that, besides the capability of neuron number determination, WNN with our initialization method gives more satisfactory and stable results for time series prediction compared to Zhang's heuristic initialization method which is used for this model in some literatures [9, 16, 17].

The paper is organized as follows. A brief review of wavelet and wavelet-based function approximation is given in Section 2, followed by the introduction of the architecture of wavelet neural network in Section 3. The detailed description of the clustering based initialization approach and the training algorithm are given in Sections 4 and 5. Three simulation experiments on time series prediction problems and the comparison results with Zhang's heuristic initialization method are presented in Section 6. Finally, some conclusions are drawn in the last section.

## 2. Wavelet-Based Function Approximation

Wavelets in the following form,

(1) $$\begin{array}{c} \psi_{a,b}={\left|a\right|}^{-1/2}\psi \left(\frac{x-b}{a}\right),\;\left(a,b\in R,a\neq 0\right), \end{array}$$

are a family of functions generated from one single function *ψ*(*x*) by the operation of dilation and translation. *ψ*(*x*) ∈ *L* ^2^(*R*) is called a mother wavelet function that satisfies the admissibility condition:

(2) $$\begin{array}{c} C_{\psi }=\overset{+\infty }{\underset{0}{\int }}\frac{{\left|\hat{\psi }\left(\omega \right)\right|}^{2}}{\omega }d\omega <+\infty , \end{array}$$

where $\hat{\psi }(\omega )$ is the Fourier transform of *ψ*(*x*) [11, 18].

Grossmann and Morlet [19] proved that any function *f*(*x*) in *L* ^2^(*R*) can be represented by

(3) $$\begin{array}{c} f\left(x\right)=\frac{1}{C_{\psi }}\iint Wf\left(a,b\right){\left|a\right|}^{-1/2}\psi \left(\frac{x-b}{a}\right)\frac{1}{a^{2}}da\;db, \end{array}$$

where *Wf*(*a*, *b*) given by

(4) $$\begin{array}{c} Wf\left(a,b\right)={\left|a\right|}^{-1/2}\overset{+\infty }{\underset{-\infty }{\int }}\psi \left(\frac{x-b}{a}\right)f\left(x\right)dx \end{array}$$

is the continuous wavelet transform of *f*(*x*).

Superior to conventional Fourier transform, the wavelet transform (WT) in its continuous form provides a flexible time-frequency window, which narrows when observing high frequency phenomena and widens when analyzing low frequency behavior. Thus, time resolution becomes arbitrarily good at high frequencies, while the frequency resolution becomes arbitrarily good at low frequencies. This kind of analysis is suitable for signals composed of high frequency components with short duration and low frequency components with long duration, which is often the case in practical situations.

As the parameters *a* and *b* are the continuous values, the resulting continuous wavelet transform (CWT) is a very redundant representation and impracticable also. This impracticability is the result of the redundancy. Therefore, the scale and shift parameters are evaluated on a discrete grid of time-scale leading to a discrete set of continuous wavelet functions:

(5) $$\begin{array}{c} \psi_{a_{i},b_{i}}={\left|a_{i}\right|}^{-1/2}\psi \left(\frac{x-b_{i}}{a_{i}}\right),\;a_{i},b_{i}\in R,\;\;a_{i}\neq 0. \end{array}$$

The continuous inverse wavelet transform (3) is discretized as

(6) $$\begin{array}{c} f\left(x\right)=\underset{i}{\sum }w_{i}{\left|a_{i}\right|}^{-1/2}\psi \left(\frac{x-b_{i}}{a_{i}}\right). \end{array}$$

If there exist two constants *c* > 0 and *C* < +*∞* such that, for any *f*(*x*) in *L* ^2^(*R*), the following inequalities hold:

(7) $$\begin{array}{c} c{\left\|f\right\|}^{2}\leq \underset{i}{\sum }{\left|\langle \psi_{a_{i},b_{i}},f\rangle \right|}^{2}\leq C{\left\|f\right\|}^{2}, \end{array}$$

where ‖*f*‖ denotes the norm of function *f*(*x*) and 〈*f*, *g*〉 denotes the inner product of functions *f* and *g*, and the family {*ψ*_*a*_*i*_,*b*_*i*__} is said to be a frame of *L* ^2^(*R*). It has been proved that families of wavelet frames of *L* ^2^(*R*) are universal approximators.

Inspired by the wavelet decomposition of *f*(*x*) ∈ *L* ^2^(*R*) in (6) and a single hidden layer network model, Zhang and Benveniste [9] had developed a new neural network model, namely, wavelet neural network (WNN).

## 3. Architecture of Wavelet Neural Network

A brief review of wavelet decomposition theory has been given in Section 2, where functions with univariable were concerned. For the modeling of multivariable processes, multidimensional wavelets must be defined. In the present work, multidimensional wavelets are defined as the multiplication of single-dimensional wavelet functions:

(8) $$\begin{array}{c} \Psi_{j}\left(x\right)=\psi \left(\frac{x-b_{j}}{a_{j}}\right)=\overset{n}{\underset{k=1}{\prod }}\psi \left(\frac{x_{k}-b_{jk}}{a_{jk}}\right),\;j=1,2,\ldots ,N, \end{array}$$

where **x** = (*x*_1_, *x*_2_,…, *x*_*n*_)^T^ is the input vector and **b**_*j*_ = (*b*_*j*1_, *b*_*j*2_,…, *b*_*jn*_) and **a**_*j*_ = (*a*_*j*1_, *a*_*j*2_,…, *a*_*jn*_) are the translation and dilation vectors, respectively.

**Figure 1:**
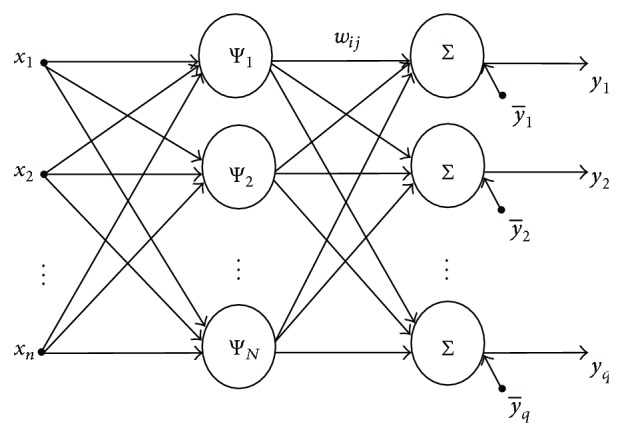
Architecture of wavelet neural network.

Generalized from radial basis function neural network, WNN is in fact a feed-forward neural network with one hidden layer, wavelet functions as activation functions in the hidden nodes, and a linear output layer. As a result, the network output **y** = (*y*_1_, *y*_2_,…, *y*_*q*_)^T^ is computed as

(9) $$\begin{array}{c} y_{i}=\overset{N}{\underset{j=1}{\sum }}w_{ij}\Psi_{j}+{\bar{y}}_{i},\;i=1,2,\ldots ,q, \end{array}$$

where **w** = (*w*_*ij*_) and $\bar{y}=({\bar{y}}_{1},{\bar{y}}_{2},\ldots ,{\bar{y}}_{q})$ define the connecting weights and the bias terms between the hidden layer and the output layer, respectively. *N* is the number of units in hidden layer. These wavelet neurons are usually referred to as wavelons. The architecture of a WNN is illustrated in Figure 1.

## 4. Initialization Approach of Wavelet Neural Network

Before training the WNN, some factors should be determined in advance, which are the number of wavelons and initial value of parameters (*a*_*jk*_, *b*_*jk*_, *w*_*ij*_, and ${\bar{y}}_{i}$). The former is fixed once the structure of network was determined, while the latter is adjusted by the training algorithm. All these factors are crucial for the performance of network in simulating the real model. In this section, a brief description of wavelet window is presented firstly, and then a novel initialization method based on the dynamic clustering is proposed, which could provide the number of hidden neurons and the initial values of translation and dilation parameters at the same time.

### 4.1. Wavelet Window in Time Domain

A mother wavelet function *ψ*(*x*) defined by (2) will have sufficient decay, which can be considered as “local response.” In other words, *ψ*(*x*) is a window with center in *μ* and radius *σ* in time domain, which can be computed by [20]

(10) $$\begin{array}{c} \mu =\overset{+\infty }{\underset{-\infty }{\int }}x{\left|\psi \left(x\right)\right|}^{2}dx,\\ \sigma^{2}=\overset{+\infty }{\underset{-\infty }{\int }}{\left(x-\mu \right)}^{2}{\left|\psi \left(x\right)\right|}^{2}dx. \end{array}$$

As a result, its translated and dilated version *ψ*_*a*,*b*_ = *ψ*((*x* − *b*)/*a*) will be concentrated in the region of [*b* + *aμ* − *aσ*, *b* + *aμ* + *aσ*] in the time domain.

**Figure 2:**
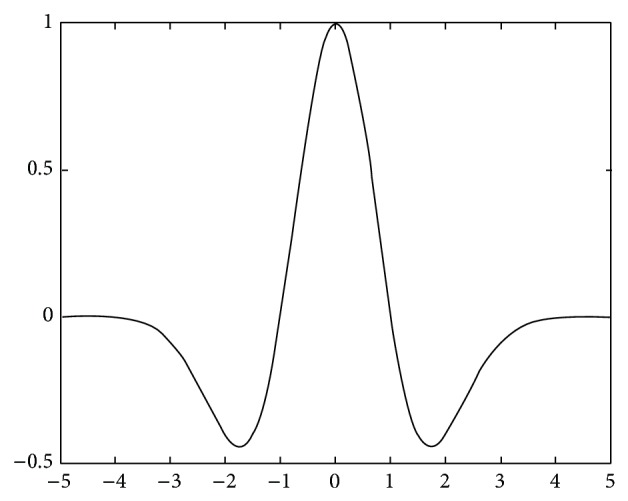
Mexican Hat wavelet.

In this paper, the Mexican Hat wavelet function with symmetric graph (Figure 2) is employed, which is given by the following equation:

(11) $$\begin{array}{c} \psi_{M}\left(x\right)={\left(1-x\right)}^{2}\cdot \exp\frac{-x^{2}}{2}. \end{array}$$

From (10), the center and radius of Mexican Hat wavelet window in the time domain can be derived as

(12) $$\begin{array}{c} \mu_{M}=0,\;\;\sigma_{M}=1.08012345. \end{array}$$

### 4.2. Initialization by a Novel Clustering Approach for WNN

The structure of our network is illustrated in Figure 1. Suppose the input data for network training are *M* vectors with *n* components: {**x**_*l*_ = (*x*_*l*1_, *x*_*l*2_,…, *x*_*ln*_)^T^, *l* = 1,2,…, *M*}. The procedure comprises the following steps:

1. Create the first cluster *C*_1_ = {**x**_1_} with cluster mean **m**_1_ = **x**_1_ and dimensional radius **r**_1_ = 0. Set the number of clusters *J* = 1.
2. Put *l* = 2.
3. Compute the distance vectors **D**_*i*_ = |**x**_*l*_ − **m**_*i*_| = (*d*_*i*1_, *d*_*i*2_,…, *d*_*in*_)^T^, *i* = 1,2,…, *J*.

If there are some **D**_*i*_*s*__ ∈ {**D**_*i*_}_*i*=1_^*J*^  (*s* = 1,2,…, *S*), such that, ∀*j* ∈ {1,2,…, *n*}, *d*_*i*_*s*_*j*_ ≤ max⁡(*r*_*i*_*s*_*j*_, th_*j*_) and Arg min⁡_*k*_(∑_*j*=1_^*n*^ *d*_*kj*_) = *K*, then **x**_*l*_ ∈ *C*_*K*_. The vector **t** **h** = (th_1_, th_2_,…, th_*n*_)^T^ is a threshold vector which is set in advance of executing the algorithm. The cluster mean will be reset as

(13) $$\begin{array}{c} m_{K}=\frac{1}{\left|C_{K}\right|}\underset{k}{\sum }x_{k} \end{array}$$

and dimensional radius **r**_*K*_ = (*r*_*K*1_, *r*_*K*2_,…, *r*_*Kn*_) will be reset as

(14) $$\begin{array}{c} r_{Kj}=\underset{k}{\max}\left|m_{Kj}-x_{kj}\right|,\;j=1,2,\ldots ,n, \end{array}$$

where |*C*_*K*_| is the cardinal number of *C*_*K*_ and **x**_*k*_ = (*x*_*k*1_, *x*_*k*2_,…, *x*_*kn*_)^T^ are the patterns that belong to *C*_*K*_.

Else, the number of clusters becomes *J* = *J* + 1; create the *J*th cluster *C*_*J*_ = {**x**_*l*_} with cluster mean **m**_*J*_ = **x**_*l*_ and dimensional radius **r**_*J*_ = 0.

(4) Put *l* = *l* + 1. If *l* > *M*, then stop; otherwise go to step (3).

**Remark 1 .**

(i) Vector **t** **h** = (th_1_, th_2_,…, th_*n*_)^T^ in the above procedure is crucial to the clustering result. Large elements of **t** **h** will lead to a coarse partition, namely, a small *J*, whereas **t** **h** with small value will lead to a large *J*. In practice, a reasonable **t** **h** should be determined by the input patterns. In our experiments, we prefer to adopt vector **t** **h** as in formula (15) to control the cluster scale, which offers moderate results in most times. Consider

(15) $$\begin{array}{c} \text{t}{\text{h}}_{j}=\sqrt{\mathrm{var}\left({\bar{x}}_{j}\right)},\;j=1,2,\ldots ,n, \end{array}$$

where ${\bar{x}}_{j}=(x_{1j},x_{2j},\ldots ,x_{Mj})$.

(ii) The conditions “there are some **D**_*i*_*s*__ ∈ {**D**_*i*_}_*i*=1_^*J*^  (*s* = 1,2,…, *S*) such that, ∀*j* ∈ {1,2,…, *n*}, *d*_*i*_*s*_*j*_ ≤ max⁡(*r*_*i*_*s*_*j*_, th_*j*_)” in step (3) are derived from “local response” property of activation wavelet functions Ψ(**x**) = ∏_*k*=1_^*n*^ *ψ*((*x*_*k*_ − *b*_*k*_)/*a*_*k*_) in the wavelons. As a result, patterns satisfying that each feature activates corresponding 1-D wavelet function will be identified as a class.

(iii) After the clustering procedure of (1)–(4), the corresponding results help us to determine the number of wavelons in WNN as *N* = *J* and the initial value of translation and dilation vectors as

(16) $$\begin{array}{c} b_{j}=m_{j}^{\text{T}}, \end{array}$$

(17) $$\begin{array}{c} a_{j}=\frac{\beta }{\sigma }r_{j}^{\text{T}}. \end{array}$$

*β* in (17) is a relaxation parameter which satisfies *β* ≥ 1; *σ* is the window radius of wavelet function *ψ*(*x*).

(iv) In order to avoid the dilation parameters being zeros, the radius vector of the cluster with single element should be redefined. The minimum value strategy is employed which can be described as *r*_*l*′*j*_ = min⁡_*l*_(*r*_*lj*_), (|*C*_*l*′_| = 1, |*C*_*l*_| > 1).

(v) The connecting weights **w** = (*w*_*ij*_) between the hidden layer and the output layer will be randomly initialized in the region [−1,1], and the bias term $\bar{y}=({\bar{y}}_{1},{\bar{y}}_{2},\ldots ,{\bar{y}}_{q})$ initialized as the mean vector of input patterns.

## 5. Training Algorithm

Gradient descent method is implemented for training the WNN in this paper. Parameters $\left(a_{jk},b_{jk},w_{ij},{\bar{y}}_{i}\right)$ are adjusted in the opposite direction of the gradient such that the objective function in (18) of the model should be minimized. Consider

(18) $$\begin{array}{c} E=\frac{1}{M}\overset{M}{\underset{l=1}{\sum }}{\left\|y_{l}-f_{l}\right\|}^{2}=\frac{1}{M}\overset{M}{\underset{l=1}{\sum }}\;\overset{q}{\underset{i=1}{\sum }}{\left(y_{li}-f_{li}\right)}^{2}, \end{array}$$

where **y**_*l*_ is the output of network and **f**_*l*_ is the desired output.

The corrections applied to parameters *a*_*jk*_, *b*_*jk*_, *w*_*ij*_, and ${\bar{y}}_{i}$ are shown as follows:

(19) $$\begin{array}{c} \Delta {\bar{y}}_{i}=-\gamma_{1}\frac{\partial E}{\partial {\bar{y}}_{i}}=-\gamma_{1}\frac{2}{M}\overset{M}{\underset{l=1}{\sum }}\left(y_{li}-f_{li}\right),\\ \Delta w_{ij}=-\gamma_{2}\frac{\partial E}{\partial w_{ij}}=-\gamma_{2}\frac{2}{M}\overset{M}{\underset{l=1}{\sum }}\left(y_{li}-f_{li}\right)\Psi_{j}\left(x_{l}\right),\\ \Delta a_{jk}=-\gamma_{3}\frac{\partial E}{\partial a_{jk}}=-\gamma_{3}\frac{2}{M}\overset{M}{\underset{l=1}{\sum }}\;\overset{q}{\underset{i=1}{\sum }}\left(y_{li}-f_{li}\right)w_{ij}\frac{\partial \Psi_{j}\left(x_{l}\right)}{\partial a_{jk}},\\ \Delta b_{jk}=-\gamma_{4}\frac{\partial E}{\partial b_{jk}}=-\gamma_{4}\frac{2}{M}\overset{M}{\underset{l=1}{\sum }}\;\overset{q}{\underset{i=1}{\sum }}\left(y_{li}-f_{li}\right)w_{ij}\frac{\partial \Psi_{j}\left(x_{l}\right)}{\partial b_{jk}}, \end{array}$$

where Ψ_*j*_(**x**_*l*_) = ∏_*k*=1_^*n*^ *ψ*((*x*_*lk*_ − *m*_*jk*_)/*d*_*jk*_), ∂Ψ_*j*_(**x**_*l*_)/∂*a*_*jk*_ = *F*(**x**_*l*_)(*x*_*lk*_ − *b*_*jk*_)(−1/*a*_*jk*_^2^), ∂Ψ_*j*_(**x**_*l*_)/∂*b*_*jk*_ = *F*(**x**_*l*_)(−1/*a*_*jk*_), and *F*(**x**_*l*_) = *ψ*′((*x*_*lk*_ − *b*_*jk*_)/*a*_*jk*_)∏_*i*=1,*i*≠*k*_^*n*^ *ψ*((*x*_*li*_ − *b*_*ji*_)/*a*_*ji*_). *γ*_1_, *γ*_2_, *γ*_3_, *γ*_4_ are the learning rates which should be set on the basis of specific experiment.

## 6. Simulation Examples

In this section, WNN model with two different initialization schemes is applied to three time series prediction problems, namely, the prediction of Mackey-Glass, Box-Jenkins, and traffic volume time series. The performance of WNN with the clustering based initialization approach (WNN-CIA) described in Section 4 is compared to Zhang's heuristic initialization approach (WNN-HIA) in each simulation.

Because the architecture of WNN-HIA must be decided in advance, in order to compare directly, we adopt the same architecture with WNN-CIA in the experiments. Relaxation parameter *β* in (17) of WNN-CIA is set as 2.5 in all simulations and the Mexican Hat function defined in (11) is employed as the wavelet function in the hidden neurons of all models. Root mean square error (RMSE) given by (20) of the training/testing set is used as index for comparing performances of WNN with different initialization schemes. Consider

(20) $$\begin{array}{c} \text{RMSE}=\sqrt{E}=\sqrt{\frac{1}{M}\overset{M}{\underset{l=1}{\sum }}\overset{q}{\underset{i=1}{\sum }}{\left(y_{li}-f_{li}\right)}^{2}}. \end{array}$$

### 6.1. Prediction of Mackey-Glass Time Series

The Mackey-Glass chaotic time series is generated from the following delay differential equation:

(21) $$\begin{array}{c} \frac{dx\left(t\right)}{dt}=\frac{ax\left(t-\tau \right)}{1+x^{10}\left(t-\tau \right)}-bx\left(t\right). \end{array}$$

Here we predict the *x*(*t* + 6) using the input variables *x*(*t*), *x*(*t* − 6), *x*(*t* − 12), and *x*(*t* − 18). Parameters in (21) are set as *a* = 0.2, *b* = 0.1, *τ* = 17, and *x*(0) = 1.2 which make the equation show chaotic behavior. One thousand input-output data points are extracted from the Mackey-Glass time series *x*(*t*), where *t* = 118 to *t* = 1117. The first 500 data pairs of the series are used as training data, while the remaining 500 data pairs are used to validate the proposed network. After performing the proposed clustering based initialization method proposed in Section 4.2, we get that the number of wavelons is *N* = 9.

**Figure 3:**
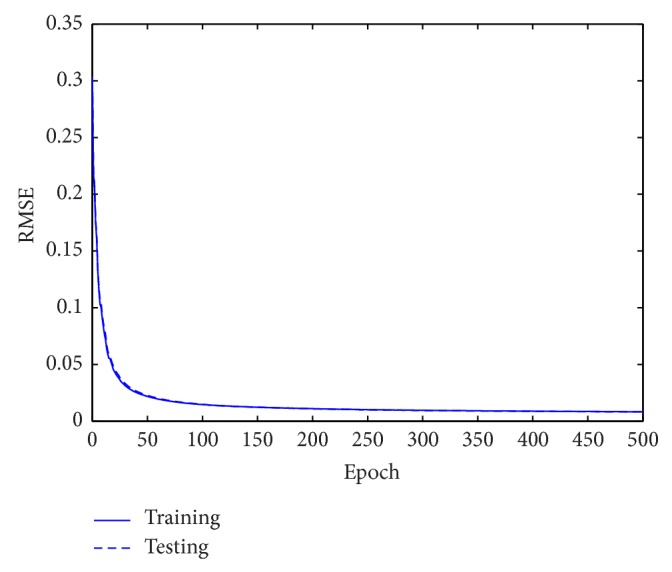
RMSE values obtained during training and testing for Mackey-Glass time series.

**Figure 4:**
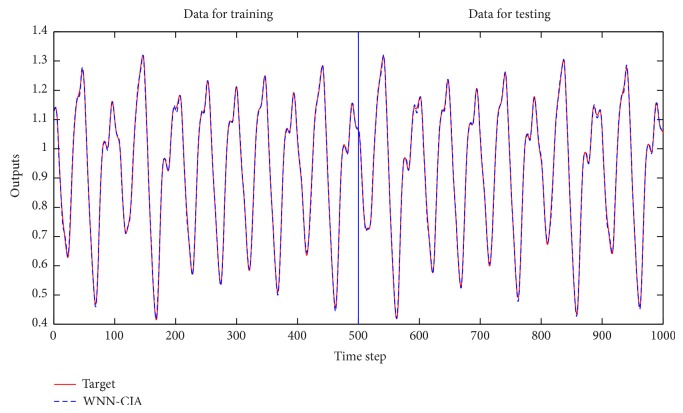
Prediction results for Mackey-Glass time series by WNN-CIA.

**Figure 5:**
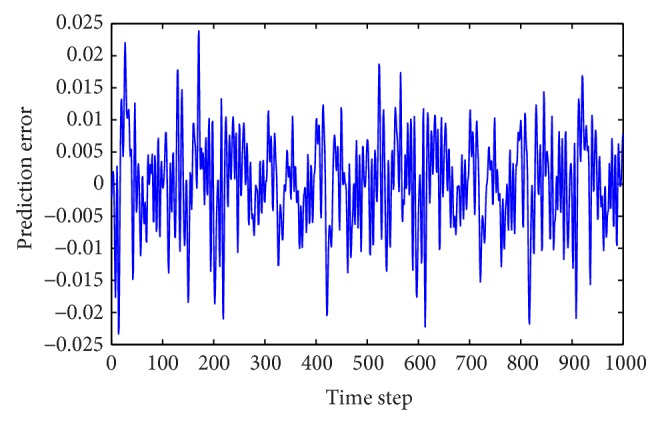
Prediction errors for Mackey-Glass time series by WNN-CIA.

For the performance comparison of WNN-CIA with WNN-HIA, some different architectures are employed for WNN-HIA.  Table 1 shows the mean and standard deviation (std.) of RMSE for training and testing data obtained when 100 runs were performed by each model. The models are trained for 500 epochs in each run. Some results of different models for testing set are shown in Table 2. The RMSE reduction curve during training and testing of gradient descent algorithm corresponding to the best WNN-CIA model is drawn in Figure 3. Figures 4 and 5 show the prediction output of the best WNN-CIA model and the corresponding prediction error for training and testing data with the training and testing RMSE as 0.0080 and 0.0078.

From Table 1, it can be seen that the performance of WNN with structure and initial parameters derived by the proposed initialization approach is much better than that of WNN-HIA, even when more parameters are employed in the model.

### 6.2. Prediction of Box-Jenkins Time Series

The gas furnace data of Box and Jenkins (1970), that is, Box-Jenkins time series, was recorded from a combustion process of a methane-air mixture. It is well known and frequently used as a benchmark example for testing identification algorithms. During the process, the portion of methane was randomly changed, keeping a constant gas flow rate. The data set consists of 296 pairs of input-output measurements. The input *u*(*t*) is the gas flow into the furnace and the output *y*(*t*) is the CO_2_ concentration in outlet gas. The sampling interval is 9 s.

In this section, the data set used consists of 292 consecutive values of methane at time (*t* − 4) and CO_2_ produced in a furnace at time (*t* − 1) as input variables, with the produced CO_2_ at time (*t*) as an output variable. Namely, variables *u*(*t* − 4) and *y*(*t* − 1) are used to predict *y*(*t*). The data are partitioned in 200 data points as a training set and the remaining 92 points as a testing set for testing the performance of the proposed network. After performing the initialization method of WNN proposed in Section 4.2, we get the number of wavelons *N* = 8.

**Figure 6:**
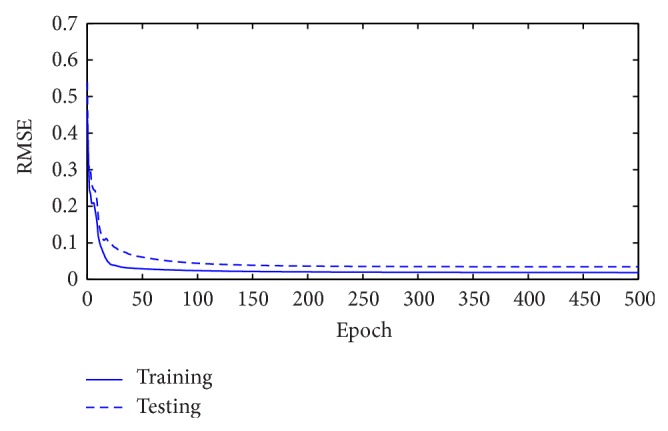
RMSE values obtained during training and testing for Box-Jenkins time series.

**Figure 7:**
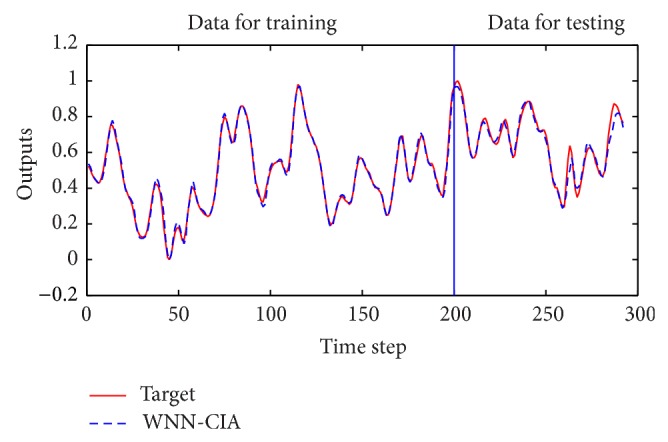
Prediction results for Box-Jenkins time series by WNN-CIA.

**Figure 8:**
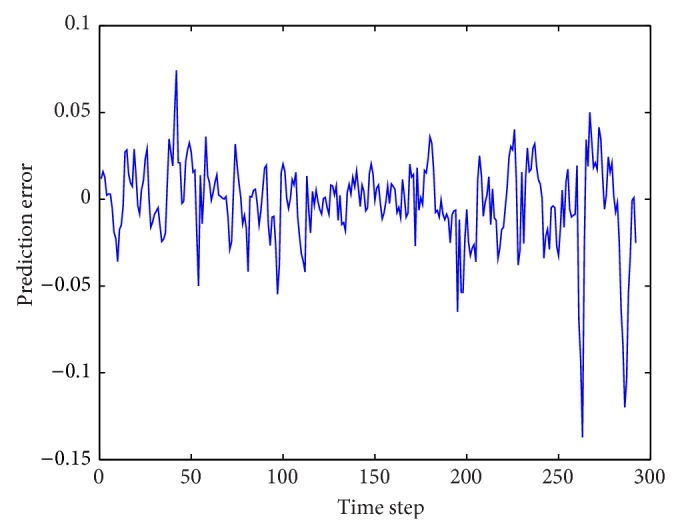
Prediction errors for Box-Jenkins time series by WNN-CIA.

As is done in Section 6.1, different architectures are employed for WNN-HIA for comparison with WNN-CIA whose structure and initial parameters are derived by the proposed approach.  Table 3 shows the mean and standard deviation of RMSE for training and testing data obtained when 100 runs were performed by each model. The models are also trained for 500 epochs in each run.  Table 4 shows some test results of different models. The RMSE reduction curve during training and testing of gradient descent algorithm corresponding to the best WNN-CIA model is drawn in Figure 6. Figures 7 and 8 show the prediction output of the best WNN-CIA model and the corresponding prediction error for training and testing data with the training and testing RMSE as 0.0186 and 0.0348.

From the data in Table 3, we can see that WNN-CIA outperforms WNN-HIA when the same architectures are employed. When more parameters are employed to WNN-HIA, the performances of WNN-HIA gradually improve. However, WNN-CIA model can make a more stable performance than all WNN-HIA models in the experiments. In order to further examine the effectiveness of the proposed method, simulation experiments of a real-word example, traffic volume time series prediction, are carried out.

### 6.3. Prediction of the Traffic Volume Time Series (A Real-Word Example)

Chen in [21] implemented the neural network time series models for traffic volume forecasting. In this section, the data of hourly traffic volume for station 5 from [21], which were collected on IR 271 and IR 90 in Cuyahoga County, are used as the real-word time series to examine the performance of WNN-CIA as well as WNN-HIA. There are 105 volume data points collected from June 4, 4:00 pm, to June 8, 12:00 pm, for training purposes, with the remaining 9 data points collected from 1:00 am to 9:00 am on June 9 reserved for model accuracy checking. This is a one-step forecasting with 6 anterior data points as input vector. Data normalizing is done to transfer values of the raw time series into the numbers in interval [0,1]. After performing the initialization method of WNN proposed in Section 4.1, we get the number of wavelons *N* = 17.

**Figure 9:**
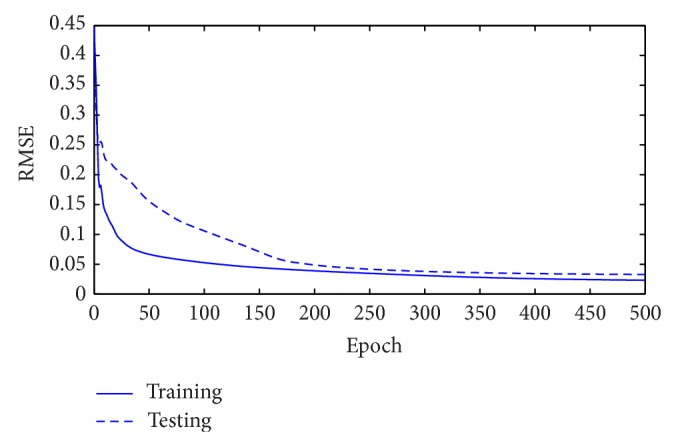
RMSE values obtained during training and testing for traffic volume time series.

**Figure 10:**
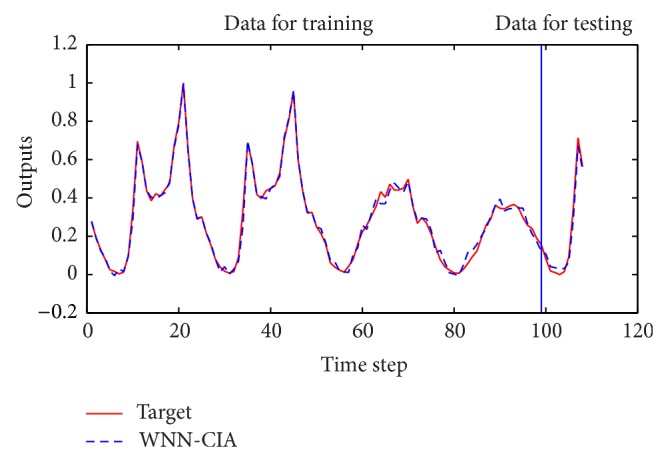
Prediction results for traffic volume time series by WNN-CIA.

**Figure 11:**
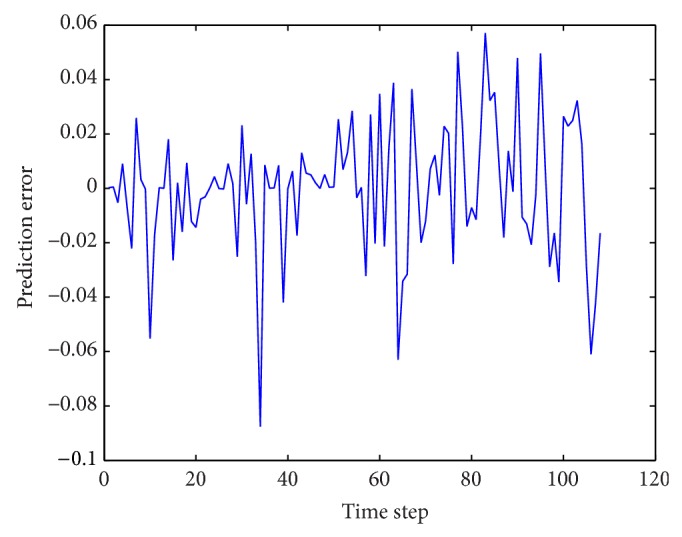
Prediction errors for traffic volume time series by WNN-CIA.

Some same and different architectures are employed for WNN-HIA for comparison with WNN-CIA. After 100 experiments with 500 epochs in each run, Table 5 shows the mean and standard deviation of RMSE for training and testing data for two WNN models with different initialization methods. Test results of different models are shown in Table 6. The RMSE reduction curve during training and testing of gradient descent algorithm corresponding to the best WNN-CIA model is drawn in Figure 9. Figures 10 and 11 show the prediction output of the best WNN-CIA model and the corresponding prediction error for training and testing data with the training and testing RMSE as 0.0233 and 0.0335.

From Table 5, we can see that the performance of WNN with the proposed clustering based initialization procedure is also superior to that with heuristic initialization approach even when more parameters are employed in WNN-HIA. It demonstrates again the validity of our methods.

## 7. Conclusion

In this paper, a novel initialization procedure for WNN as time series predictor is proposed, which behaves as a dimensional clustering procession. Taking account of the distribution of input patterns and the local response property of wavelet functions, the input patterns can be dynamically classified by the proposed approach. And then the architecture as well as the initial values of translation and dilation parameters of WNN model can be determined accordingly. Simulation results demonstrate that, besides the capability of neuron number determination, WNN with our initialization method can provide satisfactory and stable results for time series prediction.

## Figures and Tables

**Table 1:** Comparison results of WNN with two initialization methods for Mackey-Glass time series.

Model	Structure	Number of parameters	Mean of RMSE		Std. of RMSE	
			Training	Testing	Training	Testing
WNN-CIA	4-9-1	82	0.01398	0.01377	0.00188	0.00203

WNN-HIA [9]	4-8-1	73	0.02915	0.03073	0.00976	0.01079
	4-9-1	82	0.02789	0.02912	0.00899	0.00953
	4-10-1	91	0.02906	0.03059	0.01097	0.01174
	4-11-1	100	0.02700	0.02819	0.00871	0.00940

**Table 2:** Some test results of different models for Mackey-Glass time series.

Method	RMSE for testing set
Cascade correlation NN	0.06
Back-propagation NN	0.02
Sixth-order polynomial	0.04
Linear prediction method	0.55
Product T-norm [22]	0.0907
Genetic algorithm and fuzzy system [23]	0.049

**Table 3:** Comparison results of WNN with two initialization methods for Box-Jenkins time series.

Model	Structure	Number of parameters	Mean of RMSE		Std. of RMSE	
			Training	Testing	Training	Testing
WNN-CIA	2-8-1	41	0.02023	0.05900	0.00086	0.00952

WNN-HIA [9]	2-7-1	36	0.02108	0.06220	0.00121	0.01516
	2-8-1	41	0.02110	0.05964	0.00172	0.01420
	2-9-1	46	0.02068	0.05616	0.00099	0.01339
	2-10-1	51	0.02064	0.05579	0.00115	0.01064

**Table 4:** Some test results of different models for Box-Jenkins time series.

Method	Inputs	RMSE for testing set
Surmann's model [24]	2	0.400
Lee's model [25]	2	0.638
Lin's model [26]	5	0.511
Nie's model [27]	4	0.412
ANFIS model [28]	2	0.085
FuNN model [29]	2	0.071

**Table 5:** Comparison results of WNN with two initialization methods for traffic volume time series.

Model	Structure	Number of parameters	Mean of RMSE		Std. of RMSE	
			Training	Testing	Training	Testing
WNN-CIA	6-17-1	222	0.03201	0.05343	0.00462	0.01237

WNN-HIA [9]	6-16-1	209	0.03996	0.05692	0.01162	0.02125
	6-17-1	222	0.03868	0.05561	0.01109	0.02266
	6-18-1	235	0.03761	0.05373	0.01065	0.02035
	6-19-1	248	0.03796	0.05567	0.01248	0.02312

**Table 6:** Some test results of different models for traffic volume time series.

Method	Inputs	RMSE for testing set
Linear prediction method	6	0.3090
Sixth-order polynomial	6	0.1381
Back-propagation NN [21]	6	0.1244
Back-propagation NN [21]	12	0.1220
Radial basis function NN	6	0.0743
Elman NN	6	0.1041
